# The Etiology and Rational Treatment of Anasarca

**Published:** 1897-10

**Authors:** 


### FRENCH.

TRANSLATED BY DR. ALEXANDER GLASS,
PHILADELPHIA, PA.

THE ETIOLOGY AND RATIONAL TREATMENT OF ANASARCA, BY DR. CADEAC.—Anasarca is an infectious malady, occurring generally as a secondary condition. It occurs in various forms, characterized by acute dilatation of the veins and marked by petechial spots on the mucous membrane and by circumscribed œdema, with a tendency to extend over the entire body. Anasarca as a primary affection is extremely rare. It often follows a relapse in some other affection, and is localized by certain germs which occur in the blood. These germs are toxic, and produce dilatation of the venous organs. It generally follows the following diseases: Influenza, pharyngitis, acute coryza, acute laryngitis, infectious bronchitis, enteritis, gastro-enteritis, typhoid fever, abscesses, and dropsy of the extremities, accompanied by acute distention. In some cases the microbes produce suppuration, causing a toxic dilatation of the venous system, and produce anasarca. When the dilatation occurs there is an abundant collection of petechial spots. This is followed by œdema of the extremities, by hemorrhage, and finally by gangrenous portions.

The treatment of anasarca at the first is to find out the cause for the dilatation of the veins. The indications are that all antiseptic medications should be both local and general. This medication should be made with the object of lessening the production and hastening resolution of petechial spots and the œdema. The formula indicated in such a disease is 40 centigrammes of strychnia and ergotine per day. Tonics in general are to be used, especially coffee and caffeine. Anti-streptococci serum by Marmoreck is used to advantage. The only trouble about it is that this serum is very difficult to procure, but it should be always preferred as a curative agent.—*Journal de Médecine Vétérinaire, etc.*

